# Fixed differences in the 3′UTR of buffalo *PRNP* gene provide binding sites for miRNAs post-transcriptional regulation

**DOI:** 10.18632/oncotarget.17545

**Published:** 2017-05-02

**Authors:** Hui Zhao, Siqi Wang, Lixia Guo, Yanli Du, Linlin Liu, Tengfei Ma, Newton O. Otecko, Canpeng Li, Yaping Zhang

**Affiliations:** ^1^ State Key Laboratory for Conservation and Utilization of Bio-resource, Yunnan University, Kunming 650091, P.R. China; ^2^ Key Laboratory for Animal Genetic Diversity and Evolution of High Education in Yunnan Province, Yunnan University, Kunming 650091, P.R. China; ^3^ School of Life Science, Yunnan University, Kunming 650091, P.R. China; ^4^ State Key Laboratory of Genetic Resources and Evolution, Kunming Institute of Zoology, Chinese Academy of Sciences, Kunming 650223, P.R. China; ^5^ Kunming College of Life Science, University of Chinese Academy of Sciences, Kunming 650204, P.R. China; ^6^ School of Chemical Science and Technology, Yunnan University, Kunming 650091, P.R. China

**Keywords:** microRNA, difference, BSE, *PRNP*, 3′UTR

## Abstract

Bovine spongiform encephalopathy, a member of transmissible spongiform encephalopathies, has not been reported in buffaloes, *Bubalus bubalis*. Prion protein (PrP), encoded by the prion protein gene (*PRNP*), is fundamental in the pathogenesis of transmissible spongiform encephalopathies. We previously showed that cattle express more PrP proteins but lower *PRNP* mRNA than buffaloes in several pivotal tissues like the obex. Therefore, we sought to establish whether genetic variability in *PRNP* 3′UTR, mediated by miRNA down-regulation, causes PrP expression differences between cattle and buffaloes. We annotated the 3′UTR of buffalo *PRNP* gene by 3′RACE experiment. A total of 92 fixed differences in the complete 3′UTR (∼ 3 kb) were identified between 13 cattle and 13 buffaloes. Resequencing of UTR-C (g.786-1436) and UTR-B (g.778-1456) fragments confirmed that all mutations except g.1022T in cattle are fixed differences between 147 cattle and 146 buffaloes. In addition, analysis of the variation of ΔG between cattle and buffalo sequences reveals four remarkable differences. Two buffalo-specific insertion sites (a 28-bp insertion and an AG insertion in buffalo 3′UTR of *PRNP* g.970-997 and g. 1088-1089, respectively) and two mutants (g. 1007-1008 TG→CC) create compatible binding sites for miRNAs in buffalo 3′UTR. This was validated through luciferase reporter assays which demonstrated that miR-125b-5p, miR-132-3p, miR-145-5p, miR-331-3p, and miR-338-3p directly act on the fixed difference sites in buffalo 3′UTR. Additional expressional analyses show that these five miRNAs are coexpressed with *PRNP* in bovine obex tissues. Our study reveals a miRNAs regulated mechanism explaining the differences in prion expression between cattle and buffalo.

## INTRODUCTION

Transmissible spongiform encephalopathies (TSEs) are invariably fatal neurodegenerative disorders. Common examples of TSEs are bovine spongiform encephalopathy (BSE) in cattle, scrapie in small ruminants, and variant Creutzfeldt-Jakob disease (vCJD) in humans [1]. The primary pathogenic event in TSEs is conversion of a host-encoded cellular prion protein (PrP^C^) into the disease-associated isoform, PrP^Sc^, through mechanisms that remains obscure. The presence of PrP^C^ is absolutely essential for the development of TSEs, as PrP knockout mice are resistant to prion infection [2, 3]. Moreover, its high expression is associated with susceptibility and a shortened incubation time for disease development [4], and vice versa [5].

Gene expression levels can be regulated by several factors, such as variations in promoter- or repressor-binding sites with transcription factors, alterations via methylation or chromatin modifications, and miRNA regulatory processes [6]. Increasing evidences show that miRNAs are involved in diverse biological processes such as development, differentiation, proliferation, and diseases [7–9]. miRNAs are a class of small (18-24 nucleotides) endogenous and non-coding RNAs that can bind to the 3′ untranslated regions (3′UTR) of mRNAs by the RNA induced silencing complex (RISC). This alters protein expression by repressing translation or by promoting degradation of the target mRNA [10, 11]. The binding between miRNAs and target genes strictly requires maximal complementarity between a miRNA ‘seed-sequence’ (at the 5′-end of a mature miRNA, spanning 2-8 nucleotides) and the 3′UTR of the target gene [8, 10, 12]. When nucleotide mutations occur within the seed region of miRNA or within the 3′UTR complementary to the seed region, miRNA-mRNA interaction is perturbed. This would translate into reduced or inhibited post-transcriptional gene regulation, and a consequent alteration of the abundance of target gene expression [6].

Until now, BSE cases have been reported worldwide in nearly 0.2 million *Bos taurus* cattle, but never in the domestic buffalo, *Bubalus bubalis* (http://www.oie.int/en/animal-health-in-the-world/bse-specific-data/) [13]. Interestingly, previous studies have revealed genetic background differences in the BSE-associated genes between cattle and buffalo [14–16]. We also recently reported a significantly lower PrP in several tissues associated with BSE pathogenesis including cerebellum, obex, mesenteric lymph node, and bronchial lymph node, in buffalo compared to cattle [17]. Importantly, the differences in PrP expression did not correlate with corresponding *PRNP* mRNA expression. For example, compared to cattle, buffalo have significantly lower PrP expression in the obex tissue, but significantly higher *PRNP* mRNA expression [17]. The inconsistency suggests that PrP turnover may be governed by post-transcriptional regulation. However, none of the reports investigating the pathogenetic implications of genomic variation on BSE susceptibility has evaluated the role of polymorphisms in *PRNP* mRNA regulation by miRNAs.

Here, we investigated whether genetic variability within 3′UTR of *PRNP* contributes to the difference in PrP expression levels between cattle and buffalo, and whether these differences are attributable to down-regulation by miRNAs. To confirm our hypothesis, we annotated the 3′UTR region of buffalo *PRNP* by 3′RACE and sequencing. We then compared the differences in *PRNP* 3′UTR between cattle and buffalo. Buffalo-specific mutations in miRNA target sites, especially a 28-bp insertion, an AG insertion, and two mutations TG→CC, are reported. *In vitro* assays confirmed five miRNAs binding to these target sites, and their expression is validated by real-time quantitative PCR (RT-qPCR) assessment.

## RESULTS

### Differences in *PRNP* 3′UTR between cattle and buffalo

In order to annotate the *PRNP* 3′UTR of buffalo, total RNA from buffalo obex was subjected to 3′RACE experiment. The amplified products (∼ 3 kb) were observed by agarose gel electrophoresis (Supplementary Figure 1) and then cloned into a T-vector for sequencing using sequencing primers listed in Supplementary Table 1. The sequence analysis revealed that the total size of buffalo *PRNP* 3′UTR region is 3310 bp, similar to the *PRNP* 3′UTR of 3287-bp length in cattle (GenBank accession No. AJ298878).

**Figure 1:**
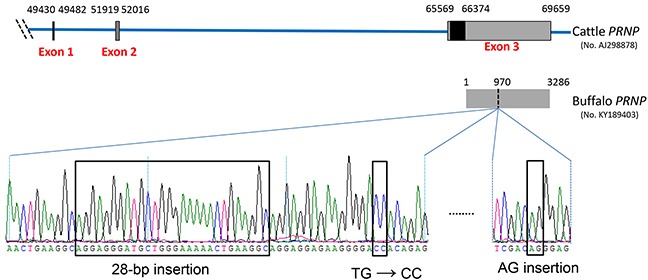
Schematic representation of the genetic structure of cattle *PRNP* and four remarkable differences in the 3′UTR of buffalo *PRNP* Nucleotide number corresponds to the *PRNP* of *Bos taurus* (GenBank accession no. AJ298878). The three boxes indicate exon 1, 2, and 3. The black box indicates the coding sequence region and is located in exon 3. The grey box in exon 3 indicates the 3′UTR of cattle *PRNP* spanning 66374 to 69659. The *PRNP* 3′UTR sequence of *Bubalus bubalis* (GenBank accession no. KY189403) was verified in this study. Compared to cattle *PRNP* 3′UTR, there are four remarkable differences in buffalo: a 28-bp insertion fragment (g. 970-997 in buffalo 3′UTR sequence), TG→CC (g. 1007-1008), and an AG-insertion (g. 1088-1089).

We then analyzed and compared the *PRNP* 3′UTR sequences cloned from 13 cattle and 13 buffaloes. As summarized in Supplementary Table 2, a total of 92 fixed differences between the two species occur in *PRNP* 3′UTR, including 85 single nucleotide polymorphisms (SNPs) and nine insertions/deletions (indel). Worth noting, a 28-bp insertion fragment is found in the 3′UTR region of buffalo *PRNP* (g. 970-997), but not in cattle sequences (Figure 1 and Supplementary Table 2). In addition, at 17-bp and 91-bp downstream of the 28-bp insertion site, there are two fixed differences, two mutations TG→CC (g. 1007-1008) and an AG-insertion (g. 1088-1089), respectively, in the buffalo *PRNP* 3′UTR (Figure 1 and Supplementary Table 2).

### The prediction of binding miRNAs by *in silico* analysis

In order to predict the fixed differences between cattle and buffalo *PRNP* 3′UTR that could affect miRNA binding, we searched miRBase [18] and utilized RNAhybrid software [19] to predict binding sites of miRNAs. Candidate miRNAs were qualified through stringent conditions described in Materials and Methods. The results show an interesting enrichment of buffalo-specific mutations overlapping the miRNA-binding sites in the region around the 28-bp insertion site. As shown in Table 1, 15 miRNAs were predicted to bind to buffalo *PRNP* 3′UTR. For example, the 28-bp insertion allele in buffalo *PRNP* 3′UTR is located in a putative binding site for six miRNAs: miR-145-5p, miR-149-5p, miR-212-5p, miR-324-3p, miR-338-3p, and miR-668-5p. Interestingly, these binding sites are not observed in cattle *PRNP* 3′UTR. Moreover, the AG insertion allele of buffalo *PRNP* 3′UTR (g. 1088-1089) was predicted to create a new target site for miR-125b-5p and miR-331-3p, which is also absent in the corresponding cattle sequence when stringent settings are applied. The seed sequence of miRNA, which spans nucleotides 2-8 at the 5′-end of mature miRNA, is important for miRNA-mRNA interaction [8]. Nucleotide alterations in the target gene complementary to the seed sequence would perturb miRNA-mRNA interaction [6]. Of note, fixed variants in buffalo *PRNP* 3′UTR occur in the regions complementary to the seed region of each of the 15 predicted miRNAs (Table 1).

**Table 1:** Differences in the 3′UTR of *PRNP* between cattle and buffalo that alter miRNA-binding potential

Predicted miRNAs	Energy	Mature miRNA sequence 5′ → 3′	Complementary sequences to seed	Buffalo		Cattle	
				Location^a^	Sequences	Sequences	Location^b^
miR-338-3p	−27.9	UCCAGCAUCAGUGAUUUUGUUGA	ATGCTGG	g.971-977	ATGCTGG	del	g.978
miR-145-5p	−25.0	GUCCAGUUUUCCCAGGAAUCCCU	AACTGGA	g.982-988	AACTGAA	del	g.978
miR-212-5p	−26.3	ACCUUGGCUCUAGACUGCUUACU	CCAAGG	g.984-990	CTGAAGG	del	g.978
miR-149-5p	−29.6	UCUGGCUCCGUGUCUUCACUCCC	AGCCAG	g.988-993	AGGCAG	del	g.978
miR-324-3p	−31.5	ACUGCCCCAGGUGCUGCUGG	GGGCAG	g.989-993	GGCAG	del	g.978
miR-668-5p	−25.4	UGCGCCUCGGGUGAGCAUG	AGGCGC	g.988-993	AGGCAG	del	g.978
miR-328-3p	−25.1	CUGGCCCUCUCUGCCCUUCCGU	GGGCCA	g.1003-1009	GGGACCA	GGGATGA	g.983-989
miR-125b-5p	−25.0	UCCCUGAGACCCUAACUUGUGA	TCAGGG	g.1087-1091	CAGGG	C- -GG	g.1067-1069
miR-331-3p	−31.9	GCCCCUGGGCCUAUCCUAGAA	CAGGGG	g.1088-1093	AGGGAG	- -GGAG	g.1068-1071
miR-27a-3p	−26.5	UUCACAGUGGCUAAGUUCCGC	CTGTGA	g.1096-1101	CTGGTG	CTGGCA	g.1074-1079
miR-668-3p	−25.4	UGUCACUCGGCUCGGCCCACUAC	AGTGAC	g.1115-1123	AGTGTCGCG	GGTGTCGCA	g.1093-1101
miR-138-1-3p	−31.0	GCUACUUCACAACACCAGGGCC	AAGTAG	g.1246-1250	AAGAG	AAGAT	g.1228-1232
miR-204-3p	−25.3	GCUGGGAAGGCAAAGGGACGU	TCCCAG	g.1328-1332	TCCCA	T- -CA	g.1310-1312
miR-132-3p	−32.5	UAACAGUCUACAGCCAUGGUCG	GACTGTT	g.1366-1370	ACTGT	TCTGT	g.1346-1350
miR-219-1-3p	−32.8	AGAAUUGUGGCUGGACAUCUG	CAATTC	g.1442-1447	CCATTC	ACATTC	g.1422-1427

^a^ and ^b^ indicate the positions in the 3′UTR sequences of GeneBank sequences for buffalo (No. KY189403) and cattle (No. KY189378), respectively.

**Figure 2:**
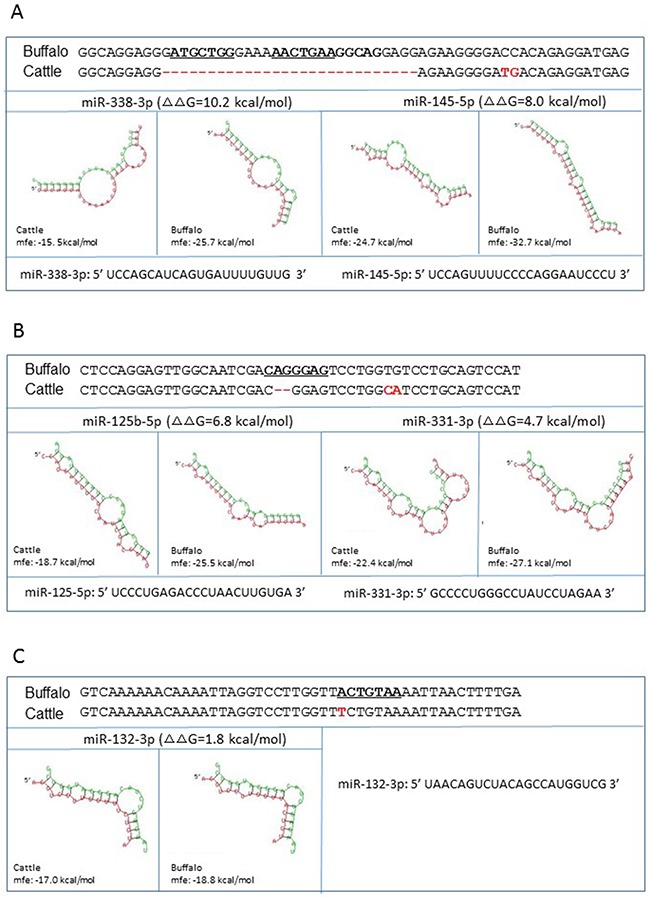
miRNA-mRNA interaction in the presence of cattle or buffalo *PRNP* allele Shown are the predicted miRNAs interact with the fragments from g.961-1020 and cattle 3′UTR g.969-1000 **(A)**, g.1067-1114 and g.1047-1092 **(B)**, and g.1338-1385 and g.1318-1365 **(C)** in buffalo and cattle PRNP 3′UTR, respectively. The Gibbs-free energy (ΔG) of miRNA-mRNA binding was measured using the RNAhybrid program. ΔΔG = ΔG _cattle_ -ΔG _buffalo_. miRNA sequence is presented in green and mRNA sequence in red. The miRNA recognition element in the buffalo mRNA are in bold and underline. The fixed differences in cattle sequence are indicated in red.

Next, we investigated the potential alterations of miRNA-mRNA interaction in the presence of cattle or buffalo *PNRP* 3′UTR with respect to each miRNA listed in Table 1. According to the method reported by Saba and co-authors [6], we retrieved seven fragments (∼50 nucleotides long) with miRNA-binding sites from buffalo *PNRP* 3′UTR, as well as their corresponding sequences from cattle *PRNP* 3′UTR (Figure 2 and Supplementary Figure 2). Subsequently, we determined the hybridization energy ΔG of miRNA-mRNA interaction using RNAhybrid program. We then calculated the variation of ΔG (ΔΔG) following the equation: ΔΔG =ΔG _cattle_ - ΔG _buffalo_, where higher ΔΔG values represent higher stability of the miRNA-mRNA duplex in buffalo sequence than in cattle. As shown in Figure 2 and Supplementary Figure 2, most cattle 3′UTR sequences show decreased miRNA binding potential except for miR-138-1-3p, compared with corresponding buffalo sequences. Relatively high ΔΔG values were detected in miR-338-3p (10.2), miR-668-5p (8.1), miR-145-5p (8.0), miR-204-3p (7.7), and miR-125b-5p (6.8), suggesting that buffalo sequences have increased binding potential to most of the miRNAs. For example, the ΔG (−32.7 kcal/mol) of buffalo 3′UTR fragment (g. 961-1020) with miR-145-5p is more negative than that of cattle (−24.7 kcal/mol), corresponding to higher stability of the miRNA-mRNA duplex (Figure 2A).

### Identification of miRNAs as regulators of buffalo *PRNP* 3′UTR

**Figure 3:**
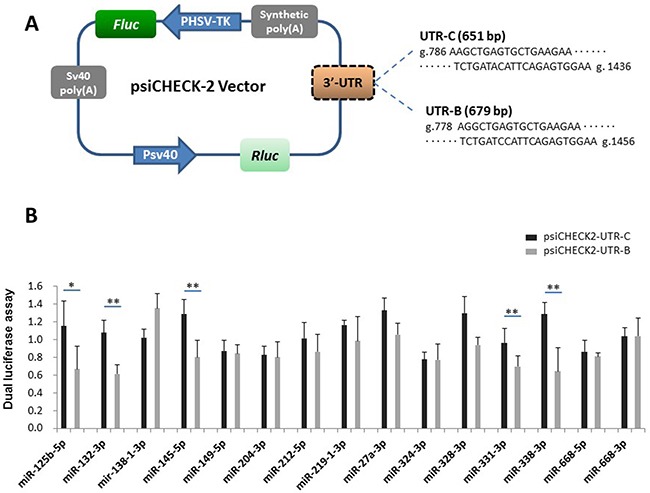
Identification of the interaction between UTR-C or UTR-B and each candidate miRNA (**A**) Diagram depicting the 3′UTR reporter constructs. The *PRNP* 3′UTR fragments of cattle (651 bp) or buffalo (679 bp) were inserted into the psiCHECK-2 vector downstream of the *Renilla* luciferase. (**B**) Luciferase assays show the effects of each miRNA on the reporter constructs containing UTR-C and UTR-B fragments. The data of the luciferase assays are presented as the mean + SD from three separate experiments. * p < 0.05; ** p<0.01.

To screen the 15 candidate miRNAs for functional effects on buffalo *PRNP*, we amplified the fragments of UTR-C and UTR-B (Supplementary Figure 1) then inserted them into psiCHECK-2 vectors (Figure 3A). The UTR-C (g.786-1436 in cattle *PRNP* 3′UTR) and UTR-B fragments (g.778-1456 in buffalo *PRNP* 3′UTR) included all the predicted miRNA-binding sites described in Table 1. We then co-transfected 293T cells with mature miRNA mimic and a psiCHECK-2 vector bearing UTR-C (psiCHECK-UTR-C) or UTR-B (psiCHECK-UTR-B) insert, or empty vector psiCHECK-2 as a control. The psiCHECK-2 dual-luciferase vector contains *firefly* luciferase and *Renilla* luciferase. *Renilla* luciferase is used to monitor changes in expression of target gene while *firefly* reporter cassette functions as an intra-plasmid transfection normalization reporter (Figure 3A). Thus, the reporter activity is evaluated using *Renilla* luciferase signal normalized to the *firefly* luciferase signal. We observed no change in reporter activity between psiCHECK2-UTR-B and psiCHECK2-UTR-C for miR-138-1-3p, miR-149-5p, miR-204-3p, miR-212-5p, miR-219-1-3p, miR-27a-3p, miR-324-3p, miR-328-3p, miR-668-5p, and miR-668-3p. However, reporter activity in psiCHECK2-UTR-B is significantly reduced for miR-125b-5p (p=0.049), miR-132-3p (p=0.009), miR-145-5p (p=0.009), miR-331-3p (p=0.034), and miR-338-3p (p=0.020), compared to psiCHECK2-UTR-C (Figure 3B). The reduction degrees (psiCHECK2-UTR-B activity/psiCHECK2-UTR-C activity) range from 50.0±2.075% by miR-338-3p to 72.6±0.701% by miR-331-3p, translating to an overall reduction degree of 59.9% by the five miRNAs. Furthermore, the conservation for mature sequences of the five miRNAs was investigated across different species, revealing high conservation among multiple species including humans, chimpanzees, mice, rats, pigs, goats, and cattle (Supplementary Figure 3). Altogether, the results suggest that buffalo allele has increased binding ability for the five miRNAs as predicted.

**Figure 4:**
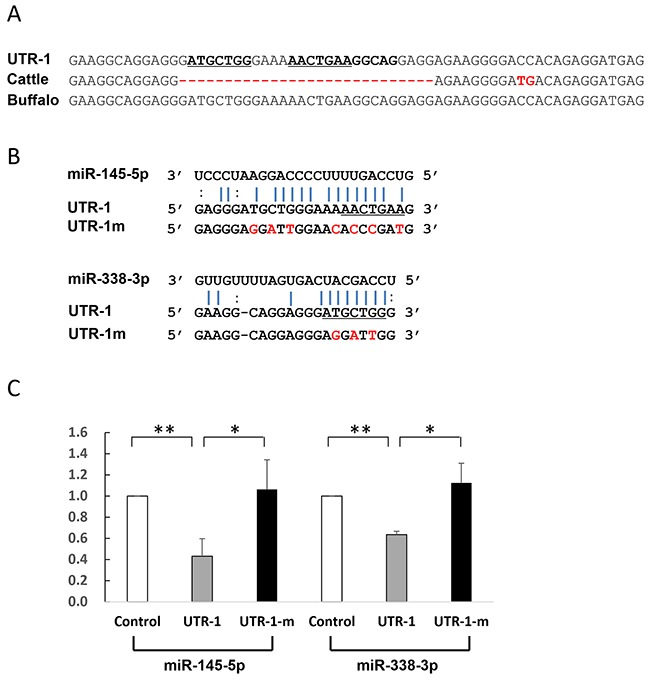
Identification of miRNAs which directly act on UTR-1 (**A**) Alignment of the UTR-1 sequences and corresponding 3′UTR sequences from cattle and buffalo. The UTR-1 sequences are from the segment of buffalo *PRNP* 3′UTR (g.958-1020), corresponding to cattle *PRNP* 3′UTR sequences g.966-1000. (**B**) Illustration shows miR-338-3p and miR-145-5p binding sites for UTR-1 and corresponding mutant binding sites for UTR-1m. (**C**) Luciferase assays show the effects of miR-338-3p and miR-145-5p on the reporter constructs containing the UTR-1 fragment. The UTR-1 or UTR-1m construct was cotransfected into 293T cells with vectors expressing miR-338-3p or miR-145-5p or miR-neg. Luciferase assay data is presented as the mean + SD from three separate experiments. * p < 0.05; ** p<0.01.

**Figure 5:**
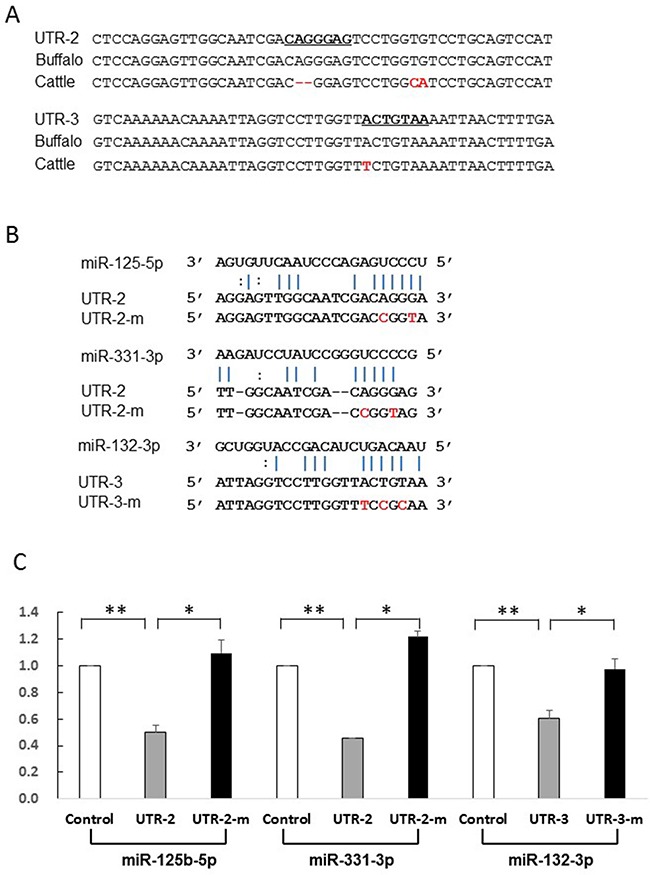
Identification of miRNAs which directly act on UTR-2 or UTR-3 (**A**) Alignment of the UTR-2 and UTR-3 sequences and corresponding the 3′UTR sequences from cattle and buffalo. The UTR-2 and UTR-3 sequences are the segment of buffalo *PRNP* 3′UTR spanning g.1067-g.1114 and g.1338-g.1385, corresponding to cattle sequence g.1047-g.1092 and g.1318-g.1365, respectively. (**B**) Illustration shows miR-125b-5p and miR-331-3p binding sites for UTR-2 and corresponding mutant binding sites for UTR-2m, miR-132-3p binding site for UTR-3 and its mutant binding sites for UTR-3m. (**C**) Luciferase assays show the effects of miR-125b-5p and miR-331-3p on UTR-2, and miR-132-3p on UTR-3 fragments. Luciferase assay data is presented as the mean + SD from three separate experiments. * p < 0.05; ** p<0.01.

Most miRNAs are thought to suppress gene expression by base pairing with the miRNA-recognition element found in their mRNA target. To identify whether the five miRNAs directly act on the putative sites, we constructed reporter vectors with insert fragments of UTR-1, UTR-2, and UTR-3 as well as their corresponding mutant seed regions UTR-1m, UTR-2m, and UTR-3m, respectively. As shown in Figure 4, the UTR-1 fragment is from buffalo *PRNP* 3′UTR g.958-1020, corresponding to cattle sequences g.966-1000. Because the UTR-1 fragment contains the 28-bp insertion site (Figure 4A), the sequences have two putative sites to respectively bind miR-338-3p and miR-145-5p. However, cattle sequence lack these binding sites (Figure 4A). Further dual-luciferase assays revealed that luciferase activity of psi-CHECK2-UTR-1 is significantly decreased in the presence of miR-145-5p (p=0.001) or miR-338-3p (p=0.004) (Figure 4C), suggesting that these miRNAs could directly decrease UTR-1 expression. To further confirm this hypothesis, we performed mutational analyses on each predicted site (Figure 4B). As expected, the mutant psi-CHECK2-UTR-1m lost sensitivity to the miRNAs (Figure 4C), indicating the specificity of the repression. In addition, UTR-2 and UTR-3 are from the segments of buffalo *PRNP* 3′UTR spanning g.1067 to g.1114, and g.1338 to g.1385, respectively, corresponding to cattle sequences from g.1047 to g.1092, and g.1318 to g.1365, respectively. The seeds of two miRNAs, miR-125b-5p and miR-331-3p, were predicted to bind to the UTR-2 at the CAGGGAG location, but cattle have no miRNA-binding site due to the deletion of AG at g.1068 site (Figure 5A, Supplementary Table 2). Moreover, the UTR-3 segment of buffalo *PRNP* 3′UTR has a binding site (ACTGTAA) for miR-132-3p, but the fixed difference (A→T in cattle g.1346) impaired the binding of miRNA to target gene (Figure 5A). As shown in Figure 5C, the luciferase activity of psi-CHECK2-UTR-2 is significantly decreased by miR-125b-5p (p=0.005) and miR-331-3p (p=0.007), and psi-CHECK2-UTR-3 by miR-132-3p (p=0.034), respectively. However, the mutant psi-CHECK2-UTR-2m and psi-CHECK2-UTR-3m vectors are unaffected by these miRNAs. Altogether, the above findings indicate that the five miRNAs can act on buffalo *PRNP* 3′UTR through the predicted binding sites.

### Resequencing

To further ascertain whether sequence variations causing differential miRNAs regulation were fixed between cattle and buffalo, we resequenced UTR-C and UTR-B fragments in 147 cattle and 146 buffaloes, respectively. Coupled with data from complete 3′UTR sequences (13 cattle and 13 buffaloes), a total of 319 sequences were obtained. We confirmed that all mutations detected during the initial screening are indeed fixed differences between the two species, except for g.1022T in cattle (Table 2). Importantly, three differences that can alter miRNA-mRNA interaction, i.e. 28-bp insertion (g. 970-997), AG insertion (g.1088-1089), and g.1366T→A, are fixed in buffalo sequences.

**Table 2:** Comparison of the genotype frequencies of mutations and indel polymorphisms in the 3′UTR of *PRNP* gene between cattle and buffalo

Cattle (160)			Buffalo (159)		
Position	Allele	Frequency	Position	Allele	Frequency
978	28-bp del	1.000	970-997	28-bp ins	1.000
987	T	1.000	1007	C	1.000
988	G	1.000	1008	C	1.000
1001	A	1.000	1021	C	1.000
1022	T	0.981	1042	C	1.000
1068	del	1.000	1088-1089	AG	1.000
1078	C	1.000	1100	T	1.000
1079	A	1.000	1101	G	1.000
1093	G	1.000	1115	A	1.000
1101	A	1.000	1123	G	1.000
1106	T	1.000	1128	C	1.000
1107	G	1.000	1129	A	1.000
1121	G	1.000	1143	C	1.000
1203	T	1.000	1221	G	1.000
1232	T	1.000	1250	G	1.000
1240	A	1.000	1258	G	1.000
1279	G	1.000	1297	T	1.000
1311	del	1.000	1329-1330	CC	1.000
1346	T	1.000	1366	A	1.000

### Spatiotemporal coexpression of miRNA-mRNA pairs

**Figure 6:**
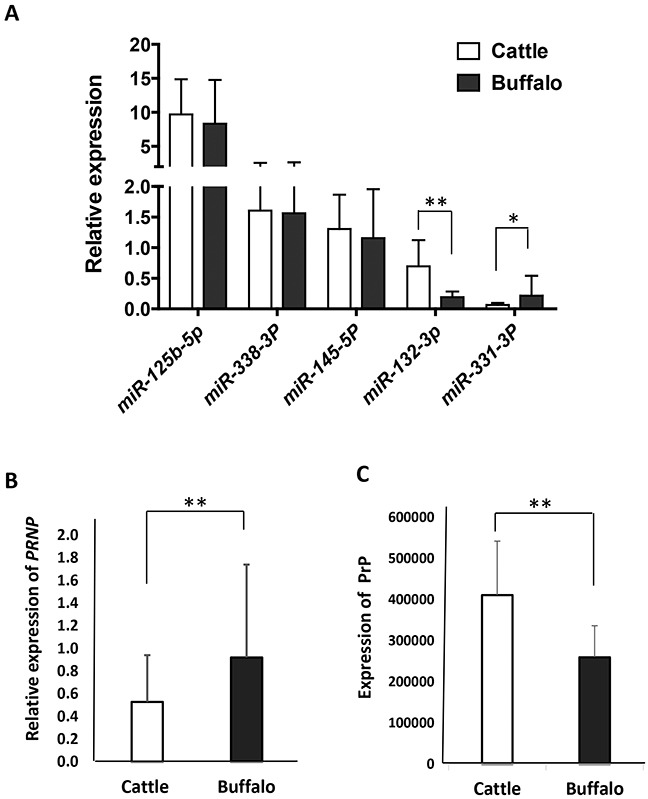
The expression of miRNAs, *PRNP* mRNA, and PrP in obex tissues from cattle and buffalo (**A**) RT-qPCR assays show the relative expression of five miRNAs in obex tissues from cattle and buffalo using U6 as an endogenous control. (**B**) RT-qPCR assays show the relative expression of *PRNP* mRNA in obex tissues from cattle and buffalo using *ACTB* as an endogenous control. (**C**) Total proteins were assessed in Western blot experiments, and the relative PrP was measured by normalizing using actin as a control.

It is a prerequisite for both miRNA and its target gene to be coexpressed in a tissue where they exert a given biological function [20]. Since obex tissues may be involved in post-transcriptional regulation [17], we conducted RT-qPCR to assess the coexpression of each miRNA/*PRNP* gene in obex tissues sampled from 28 cattle and 25 buffaloes. As shown in Figure 6, the five miRNAs are expressed in both cattle and buffalo obex tissues, indicating that they are potential functional targets in bovine obex. The highest expression occurs in miR-125b-5p, while miR-331-3p has the lowest expression. The expression of miR-132-3p is significantly higher in cattle (p=0.001) than in buffalo, while miR-331-3p is more highly expressed in buffalo than in cattle (p=0.015). In addition, buffalo express significantly higher *PRNP* mRNA (p=0.032) and lower PrP (p <0.001) than cattle in obex tissues (Figure 6), mirroring, but based on a larger sample, previously reported pattern [17].

## DISCUSSION

Buffaloes are ruminants belonging to the same phylogenetic family as cattle but having longer lifespan. Paradoxically, unlike in cattle, no BSE case has been reported from the large buffalo populations distributed worldwide. For instance, Italy has a 120,000 head-count and no history of BSE in their buffalo population, whereas 48 BSE cases were detected in cattle in 2001 (http://www.oie). This situation portrays a complex pathophysiological trajectory for TSEs.

In the present study, we identified a new mechanism to explain the difference in prion expression which is potentially associated with BSE susceptibility between cattle and buffalo. PrP plays a curial role in the development and transmission of TSEs. Many nonsynonymous coding changes in PrP have been associated with susceptibility and development of TSEs [21, 22]. Variants within regulatory regions, which could result in alterations of gene expression levels, have also been associated with BSE-susceptibility [6, 23–27]. Particularly, expression regulation by miRNAs is an important mode of post-transcriptional gene regulation. miRNAs inhibit the expression of their target mRNA through translational repression, mRNA cleavage, or mRNA deadenylation, depending on the degree of complementarity between miRNA and target gene [28]. In most animals, the miRNAs are imperfectly complementary to their target genes, making translational repression the predominant mechanism for miRNA action in animal cells [29]. Therefore, we hypothesized that genetic variation of *PRNP* 3′UTR between cattle and buffalo may affect miRNA targeting considering that PrP expression is not correlated with corresponding *PRNP* mRNA expression between cattle and buffalo (Figure 6) [17].

To confirm our hypothesis, we compared the differences in *PRNP* 3′UTR between cattle and buffalo and found a total of 91 fixed differences (Tables 2 and Supplementary Table 2). A remarkable finding in our study is the identification of a buffalo-specific 28-bp insertion site in *PRNP* 3′UTR (Figure 1), providing potential binding sites to six miRNAs (Table 1). Of these miRNAs, miR-338-3p and miR-145-5p were further confirmed to regulate buffalo-specific targets through binding to the 28-bp insertion sites (Figures 3 and 4). Additionally, the buffalo-specific AG insertion and g.1346 T→A mutation provide binding sites for miR-125b-5p and miR-331-3p, and miR-132-3p, respectively (Table 1). Further functional experiments verified that these miRNAs could mediate buffalo *PRNP* gene silencing by binding to the predicted sites (Figures 3 and 5). These results strongly support our hypothesis that variants in buffalo *PRNP* 3′UTR creating binding sites for miRNA contribute to lower PrP expression in buffalo compared to cattle.

Additionally, *in silico* predictions are useful tools for calculating alterations of miRNA-mRNA interactions in the presence of cattle or buffalo 3′UTR sequences. In general, higher ΔΔG values indicate higher stability of miRNA-mRNA duplex. The regulatory differences between cattle and buffalo sequences were further confirmed by *in vitro* assays in miR-338-3p (ΔΔG=10.2 kcal/mol), miR-145-5p (ΔΔG=8.0 kcal/mol), miR-125b-5p (ΔΔG=6.8 kcal/mol), miR-331-3p (ΔΔG=4.7 kcal/mol), and miR-132-3p (ΔΔG=1.8 kcal/mol). However, *in vitro* confirmatory tests were not perfectly consistent with the *in silico* evaluation in other ten miRNAs (Figures 2 and 3), especially in miR-668-5p and miR-204-3p (Supplementary Figure 2), despite high ΔΔG values (8.1 and 7.7 kcal/mol, respectively). Additionally, no regulatory variant was detected in the confirmatory test. Therefore, we agree with the recommendation of Saba and co-authors [6] that ascertaining the true biological function of 3′UTR SNP-miRNA interactions is a critical requirement.

Previous studies of miRNA expression profiles have reported enrichment of several miRNAs including miR-125b, miR-132-3p, miR-331, and miR-338-3p in mammalian brains [30, 31]. Similarly, the four miRNAs and miR-145-5p, particularly miR-125b-5p, miR-338-3p, and miR-145-5p, were confirmed to have high expression levels in both cattle and buffalo obex tissues (Figure 6A). Of the five miRNAs, miR-331-3p showed significantly higher expression in buffalo obex tissues than in cattle (Figure 6A). This observation suggest that variants located at the target-binding sites of these miRNAs could more greatly impact the regulation of *PRNP* gene as compared to variants in miRNAs expressed at lower levels.

Aberrant miRNA expression patterns are involved in prion diseases [32–36]. However, it is unknown whether aberrant miRNA expression directly triggers the development of prion diseases. Alternatively, various disease processes could induce miRNA deregulation, which then activate targets in biological pathways such as intracellular protein-degradation, cell death related signaling pathways, synapse function and neurogenesis, cholesterol homeostasis, and synaptoneurosomal function [33, 35, 36]. Among the deregulated miRNAs in our study is miR-338-3p, which previously showed over 2.5 fold down-regulation after infection with PrP^Sc^ [36]. miR-338-3p was capable of direct binding to buffalo *PRNP* 3′UTR in our study (Figures 3 and 4). Additionally, findings about miR-132 and miR-125b-5p are of high interest because their aberrant expression is involved in neurodegenerative diseases such as Alzheimer's disease (AD) [37–39] and Huntingtong's diseases [40, 41]. Particularly, miR-132 is associated with CNS physiology including neuronal migration and integration, dendritic outgrowth and complexity, axon outgrowth and guidance, synaptogenesis, and synaptic plasticity [42–50]. Taken together, these findings suggested that the miRNAs are implicated in many neurodegenerative diseases.

## CONCLUSION

To the best of our knowledge, the present study is the first systematic research on bovine *PRNP* post-transcriptional regulation by miRNAs. We detected multiple fixed differences between the *PRNP* 3′UTR of cattle and buffalo. Fixed differences in buffalo lead to direct binding between miRNAs and *PRNP* mRNA. We highlight the possibility that genetic variants of the *PRNP* 3′UTR can account for PrP expression differences between cattle and buffalo through miRNAs regulation. These differences may contribute to different BSE risk patterns between the two species.

## MATERIALS AND METHODS

### Animals and samples

For the whole 3′ UTR region sequencing, a total of 26 samples including 13 cattle and 13 buffaloes were used. Further sequencing of fragments of interest was performed in 319 samples. These samples included 160 cattle covering six different breeds (Dali, Fujian, Hasake, Wenshan, Xishuangbanna, and Zhaotong) and 159 buffaloes covering six different breeds (Chongqing, Fujian, Guangdong, Guangnan, Guangxi, and Xishuangbanna). Animals were maintained and experiments conducted in accordance with the guidelines of Beijing Municipality on the Review of Welfare and Ethics of Laboratory Animals. Approval was obtained from the Ethics and Experimental Animal Committee of Kunming Institute of Zoology, Chinese Academy of Sciences.

### 3′ rapid-amplification of cDNA ends (RACE)

Total RNA from buffalo obex tissue was isolated using the RNeasy^®^ Lipid Tissue Kit (Qiagen, German). Total RNA was reverse-transcribed into complementary DNA (cDNA), followed by 3′RACE experiment using the 3′Full RACE Core set with PrimeScript TM RTase (Takara Biotechnology, Dalian, China). The 3′UTR sequences between cattle (GenBank accession No. AJ_298878) and sheep (GenBank accession No. NC_019470.2) were compared and the conserved regions selected to design suitable primers. The 3′RACE-PCR was performed using specific forward primer 3′RACE_PCR_F and reverse primer 3′RACE_PCR_R and subsequent sequencing performed using sequencing primers listed in Supplementary Table 1. Sequence analysis was performed with an ABI PRISM 3700 DNA sequencer according to the manufacturer's instructions. Resulting *PRNP* 3′UTR sequences were deposited in GenBank under accession no. KY189403.

### Sequence analysis

The whole 3′UTR region of bovine *PRNP* gene was amplified by PCR using the primer pairs: 5′-CCGAAACTGACATCAAGAT-3′ (forward) and 5′-TCCAGAATGCAAGGACTG-3′ (reverse) for cattle, and 5′-CCGAAACTGACATCAAGAT-3′ (forward) and 5′-CAAATGAAGCTGCCAACA-3′ (reverse) for buffalo. The amplicons were then sequenced using sequencing primers listed in Supplementary Table 1. The UTR-C (g.786-1436 in cattle, 651-bp in length) and UTR-B (g. 778-1456 in cattle, 679-bp in length) fragments were amplified and sequenced using the primer pairs: 5′-AAGCTGAGTGCTGAAGAA-3′ (forward) and 5′-TTCCACTCTGAATGTATC-3′ (reverse). Where sequences were heterozygous for indel polymorphism, the amplified PCR products were cloned into PMD 18-T vector (Takara, Dalian, China). Five clones containing insertions were sequenced with T-vector-F and T-vector-R primers (Supplementary Table 1). Resulting sequences were deposited in GenBank under accession Nos: KY189356 - KY189378, and KY189379 - KY189403 for the complete *PRNP* 3′UTR of cattle and buffalo, respectively, as well as KY189404 - KY189549 and KY189550 - KY189696 for UTR-B and UTR-C fragments, respectively.

### miRNAs prediction

The UTR-C and UTR-B fragments were used to predict binding of miRNAs using miRBase (http://www.mirbase.org/) and RNAhybrid software (http://bibiserv.techfak.uni-bielefeld.de/rnahybrid/). miRNAs simultaneously matching the following conditions were selected as candidates for dual luciferase reporter assay: 1) binding-free energy of miRNA and target duplex below -25 kcal/mol, 2) high conservation in seed regions among mammals, 3) alteration of potential miRNA-binding due to the fixed differences between the two species, and 4) expression in brain reported in previous studies [31, 51, 52].

### Generation of 3′UTR reporter constructs of *PRNP*

The UTR-C and UTR-B fragments of bovine *PRNP* were amplified using primers: 5′-CTCGAGAAGCTGAGTGCTGAAGAA-3′ (forward) and 5′-GCGGCCGCTTCCACTCTGAATGTATC-3′ (reverse). The primers contained XhoI and NotI restriction sites (underlined nucleotides). For target identification, DNA oligos of UTR-1, UTR-2, and UTR-3, and their corresponding mutations within 7-8 nt seed-matched sites (UTR-1-m, UTR-2-m, and UTR-3-m) were synthesized as described in Supplementary Table 3. These amplicons and oligos were cloned into XhoI/NotI-digested psiCHECK-2 vector (Promega, Madison, WI). Generated constructs were confirmed by direct sequencing using the psiCHECK-2 vector sequencing primer pairs: 5′-GCAACTACAACGCCTACCTTCGG-3′ (forward) and 5′-CGAAAAGGTCACACTCTGGGGCG-3′ (reverse).

### Dual luciferase reporter assays

293T cell line was obtained from Invitrogen (Carlsbad, CA). Cells were grown in Dulbecco's Modified Eagle's Medium (DMEM) supplemented with 10% fetal bovine serum and antibiotics (100 U/ml penicillin and 100 μg/ml streptomycin). The culture media and fetal bovine serum were purchased from GE (Utah, USA) and Gibco (Australia), respectively. Cells were seeded into 48-well plates, and 70,000 cells/well were reverse transfected using lipofectamineTM2000 (Invitrogen). psiCHECK-2 derived reporter plasmids and synthesized miRNA mimics or miR-neg (GenePahma, Shanghai, China) were co-transfected at a ratio of 0.05: 0.75 (μg). After transfection for 48 hours, cells were harvested and *Firefly* and *Renilla* luciferase activities measured sequentially using the Dual-Luciferase® Reporter Assay System (Promega, Madison, WI) on GloMax™ 96 Microplate Luminometer (Promega, Madison, WI).

### Quantitative reverse transcription polymerase chain reaction (RT-qPCR) analysis

Total RNA from obex tissues of 53 animals (28 cattle and 25 buffaloes) were used for miRNA expression analysis. Total RNA (2 μg) was reverse-transcribed using miScript II RT Kit (Qiagen, Germany) with miScript HiSpec buffer to obtain cDNA. qPCR was performed to assess miRNA levels using ABI PRISM 7500 Real-Time system (Applied Biosystems, Foster, CA, USA). Primers for miRNAs amplification were purchased from miScript primer assay (Qiagen, USA). All qPCR reactions were carried out with 20-μL final volume, containing 1×QuantiTect SYBR Green PCR Master Mix (Qiagen, Germany), 2 μL of each PCR primer, and 1μL cDNA. Reaction conditions were as follows: 95°C for 15 min, followed by 40 cycles of 94°C for 15 s, 55°C 30 s and 70°C for 30 s. Each reaction was performed in triplicate. The ΔΔCt method was used to compare the relative expression levels using U6 snRNA as an internal control.

### Western blot

The above mentioned animals were also used to analyze prion expression from obex tissues by Western blot as previously described [17].

### Analysis software

The lane profile densitometry obtained from the Western blot data was analyzed using AlphaView software (www.proteinsimple.com). Two-tail student's *t* tests were used to determine statistical significance using SPSS 22.0 software (IBM).

## SUPPLEMENTARY FIGURES AND TABLES






