# Supplementary material for: Fixed differences in the 3′UTR of buffalo PRNP gene provide binding sites for miRNAs post-transcriptional regulation
Source: Oncotarget. 2017 May 2;8(28):46006–19. doi: 10.18632/oncotarget.17545 (PMC5542244; doi:10.18632/oncotarget.17545)
Supplement: Supplementary file 1 [file oncotarget-08-46006-s001.pdf]

Fixed differences in the 3'UTR of buffalo *PRNP* gene provide binding sites for miRNAs post-transcriptional regulation

SUPPLEMENTARY MATERIALS

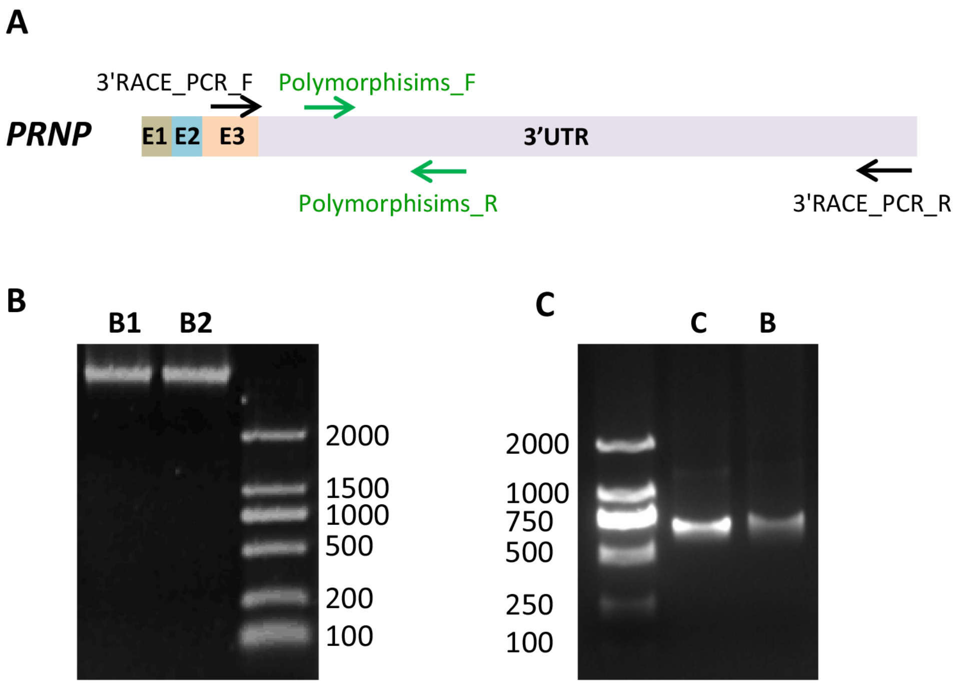

Supplementary Figure 1: PCR to amplify the 3′UTR of buffalo *PRNP* and the fragments of UTR-C and UTR-B. (**A**) The primer pair of 3′RACE_PCR_F and 3′RACE_PCR_R was developed to amplify the 3′UTR of buffalo *PRNP*. The primers of Polymorphisims_F and Polymorphisims_R were used to amplify the fragments of UTR-C and UTR-B for cattle and buffalo *PRNP*, respectively. (**B**) Representative PCR results for the 3′UTR of buffalo *PRNP* from two buffalo obex tissues (B1 and B2) are shown. (**C**) The PCR amplicons of UTR-C and UTR-B are presented in agarose gel.

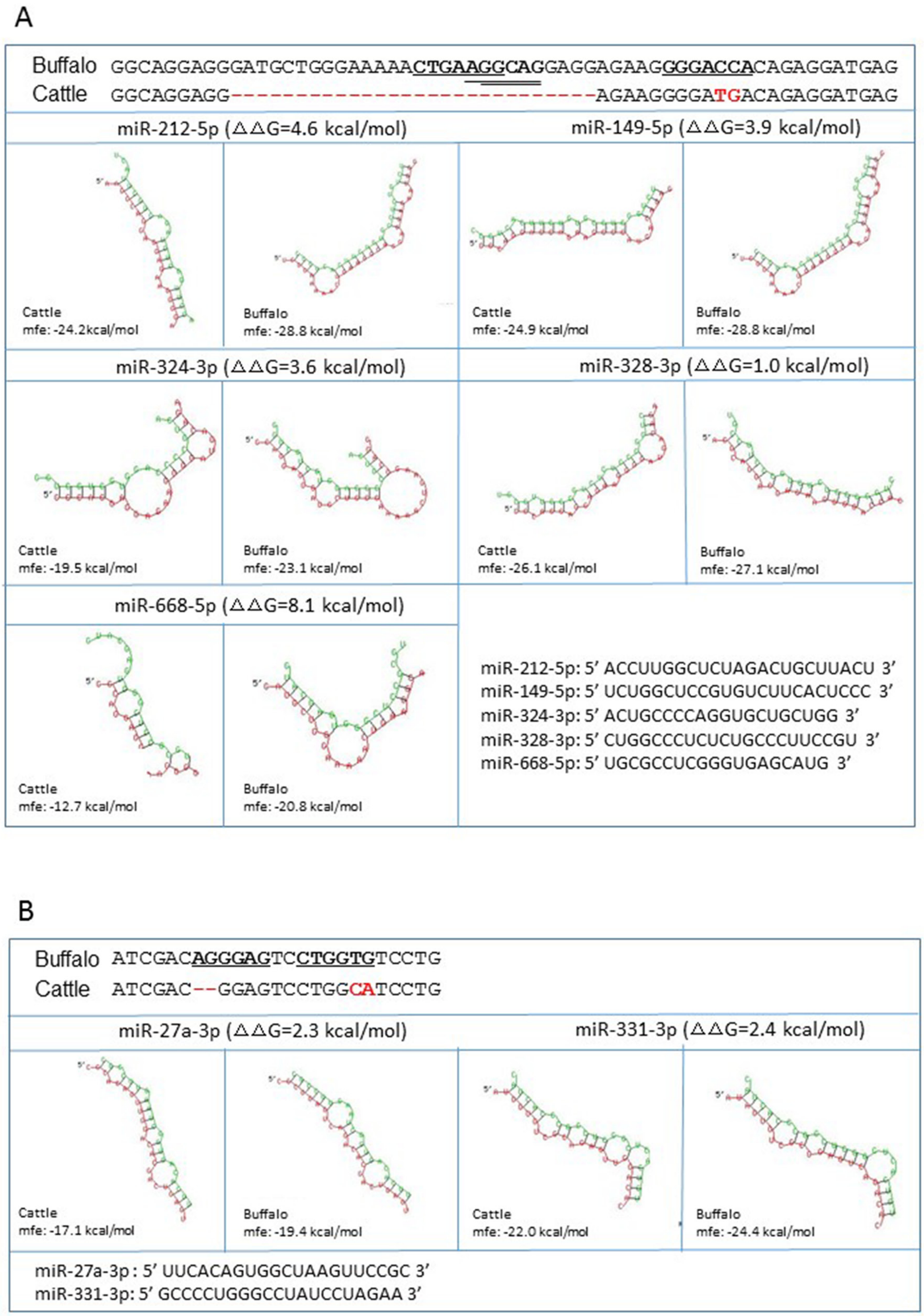
A
Buffalo GGCAGGAGGGATGCTGGGAAAAACTGAAGGCAGGAGGAGAAGGGGACCACAGAGGATGAG
Cattle GGCAGGAGG--------------------------AGAAGGGGATGACAGAGGATGAG
miR-212-5p (△△G=4.6 kcal/mol)
miR-149-5p (△△G=3.9 kcal/mol)
Cattle
mfe: -24.2kcal/mol
Buffalo
mfe: -28.8 kcal/mol
Cattle
mfe: -24.9 kcal/mol
Buffalo
mfe: -28.8 kcal/mol
miR-324-3p (△△G=3.6 kcal/mol)
miR-328-3p (△△G=1.0 kcal/mol)
Cattle
mfe: -19.5 kcal/mol
Buffalo
mfe: -23.1 kcal/mol
Cattle
mfe: -26.1 kcal/mol
Buffalo
mfe: -27.1 kcal/mol
miR-668-5p (△△G=8.1 kcal/mol)
Cattle
mfe: -12.7 kcal/mol
Buffalo
mfe: -20.8 kcal/mol
miR-212-5p: 5′ ACCUUGGCUCUAGACUGCUUACU 3′
miR-149-5p: 5′ UCUGGCUCCGUGUCUUCACUCCC 3′
miR-324-3p: 5′ ACUGCCCCAGGUGCUGCUGG 3′
miR-328-3p: 5′ CUGGCCCUCUCUGCCCUUCCGU 3′
miR-668-5p: 5′ UGCGCCUCGGGUGAGCAUG 3′
B
Buffalo ATCGACAGGGAGTCCTGGTGTCCTG
Cattle ATCGAC--GGAGTCCTGGCATCCTG
miR-27a-3p (△△G=2.3 kcal/mol)
miR-331-3p (△△G=2.4 kcal/mol)
Cattle
mfe: -17.1 kcal/mol
Buffalo
mfe: -19.4 kcal/mol
Cattle
mfe: -22.0 kcal/mol
Buffalo
mfe: -24.4 kcal/mol
miR-27a-3p : 5′ UUCACAGUGGCUAAGUUCCGC 3′
miR-331-3p: 5′ GCCCCUGGGCCUAUCCUAGAA 3′

(*Continued*)

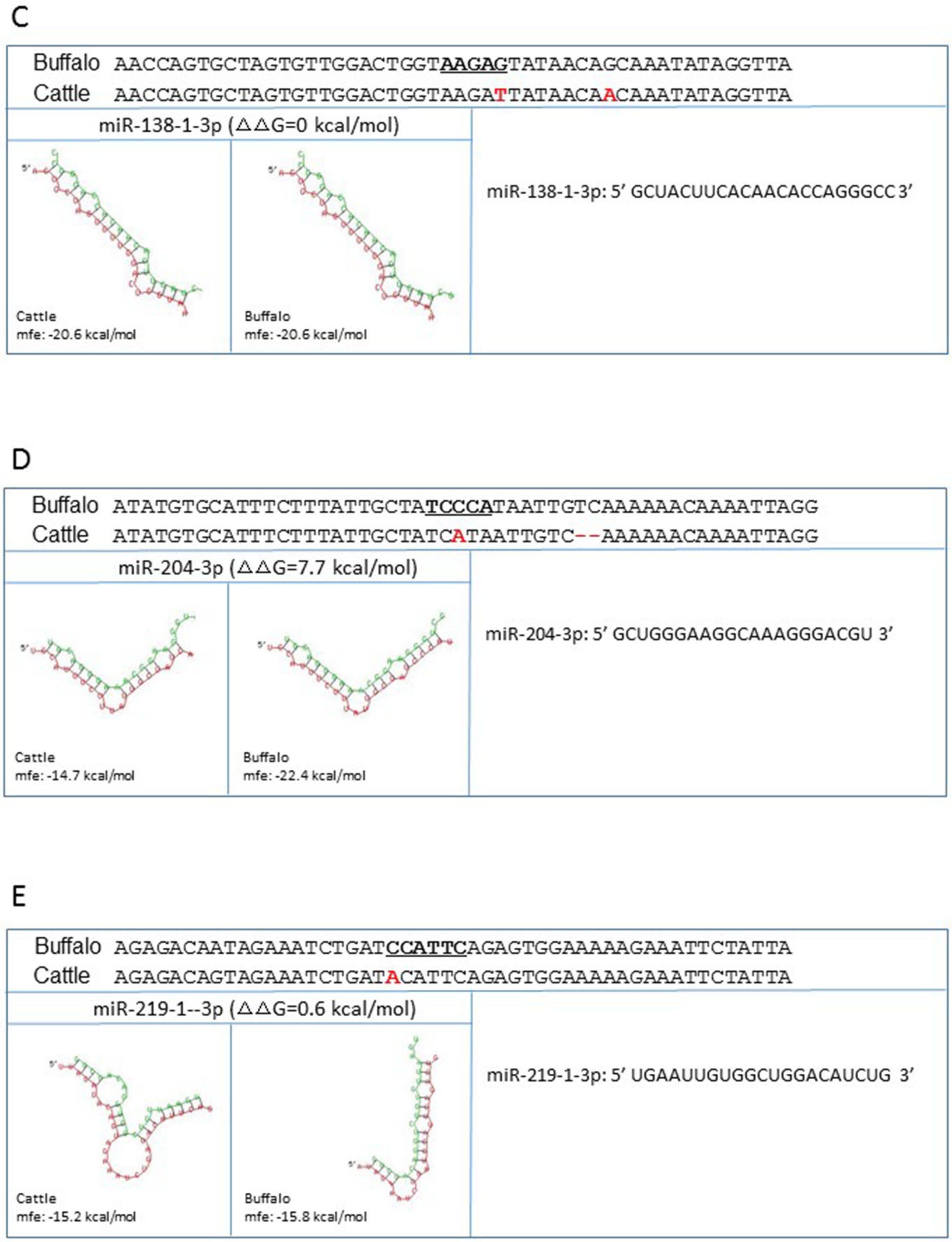

Supplementary Figure 2: The miRNA-mRNA interaction in the presence of cattle or buffalo *PRNP* allele. Shown are the predicted miRNAs interact with the fragments from g.961-1020 and g.969-1000 **(A)**, g.1082-1106 and g.1062-1084 **(B)**, g.1222-1271 and g.1204-1253 **(C)**, g.1305-1356 and g.1287-1336 **(D)**, and g.1422-1471 and g.1402-1451 **(E)** in buffalo and cattle PRNP 3'UTR, respectively. The Gibbs-free energy (ΔG) of miRNA-mRNA binding was measured using the RNAhybrid program. $\Delta\Delta G = \Delta G_{cattle} - \Delta G_{buffalo}$. The miRNA sequence is presented in green and the mRNA sequence in red. The miRNA recognition element in the buffalo mRNA are in bold and underline. The fixed differences in cattle sequence are indicated in red.

has-miR-125b-5p	ucccugagacccuaacuuguga
mmu-miR-125-5p	ucccugagacccuaacuuguga
rno-miR-125-5p	ucccugagacccuaacuuguga
ssc-miR-125	ucccugagacccuaacuuguga
chi-miR-125-5p	ucccugagacccuaacuugu - -
bta-miR-125	ucccugagacccuaacuuguga

has-miR-132-3p	uaacagucuacagccauggucg
mmu-miR-132-3p	uaacagucuacagccauggucg
rno-miR-132-3p	uaacagucuacagccauggucg
ssc-miR-132	uaacagucuacagccauggucg
bta-miR-132	uaacagucuacagccauggucg

has-miR-145-5p	guccaguuuucccaggaaucccu
mmu-miR-145-5p	guccaguuuucccaggaaucccu
rno-miR-145-5p	guccaguuuucccaggaaucccu
ssc-miR-145-5p	guccaguuuucccaggaaucccu
chi-miR-145-5p	guccaguuuucccaggaaucccu
bta-miR-145	guccaguuuucccaggaaucccu

has-miR-331-3p	gccccugggccuauccuagaa -
mmu-miR-331-3p	gccccugggccuauccuagaa -
rno-miR-331-3p	gccccugggccuauccuagaa -
ssc-miR-331-3p	gccccugggccuauccuagaa -
chi-miR-331-3p	- ccccugggccuauccuagaac
bta-miR-338	gccccugggccuauccuagaa -

has-miR-338-3p	uccagcaucagugauuuuguug -
mmu-miR-338-3p	uccagcaucagugauuuuguug -
rno-miR-338-3p	uccagcaucagugauuuuguuga
ssc-miR-338	uccagcaucagugauuuuguug -
chi-miR-338-3p	uccagcaucagugauuuuguu - -
bta-miR-338	uccagcaucagugauuuuguuga

Supplementary Figure 3: The evolutional conservation of five miRNAs. The miRNA sequences were downloaded from miRBase (http://www.mirbase.org/). has-: human; mmu-: mouse; rno-: rat; ssc-: pig; chi-: goat; bta-: cattle.

Supplementary Table 1: Primers used for amplifying and sequencing the 3′UTR of *PRNP* gene in buffalo

PCR Primers	Primer sequences (5′→3′)	Position [a]	Orientation
3′RACE_PCR_F	AAGATGATGGAGCGAGTG		F
3′RACE_PCR_R	TGGAGTGGGTTGCTATTT		R
Sequencing Primer_1	AAGATGATGGAGCGAGTG	66221	F
Sequencing Primer_2	ATCCTTAGTTCATAGACCC	66720	F
Sequencing Primer_3	TTCCACTCTGAATGTATC	67808	R
Sequencing Primer_4	AAGCTGAGTGCTGAAGAA	67158	F
Sequencing Primer_5	AGTATTGTCACCTAGCAGAT	67863	F
Sequencing Primer_6	GAGCATTGTCACCTAGCAGAT	68580	R
Sequencing Primer_7	TTTTGATGAACAGTGGATAC	68860	F
Sequencing Primer_8	TGAATACAGCAGGTGAAT	69197	R
T-vector_sequencing primer	CGCCAGGGTTTTCCCAGTCACGAC		F
T-vector_sequencing primer	AACAGGCTATGACCATG		R

[a] Indicates position in sequence AJ298878 of GeneBank.

Supplementary Table 2: Overview of the fixed differences in the 3′UTR of *PRNP* gene between cattle and buffalo

See Supplementary file 1

Supplementary Table 3: Target segment fixed point mutation of *PRNP* 3′UTR

See Supplementary file 2
