# Supplementary material for: Fixed differences in the 3′UTR of buffalo PRNP gene provide binding sites for miRNAs post-transcriptional regulation
Source: Oncotarget. 2017 May 2;8(28):46006–19. doi: 10.18632/oncotarget.17545 (PMC5542244; doi:10.18632/oncotarget.17545)
Supplement: Supplementary file 2 [file oncotarget-08-46006-s002.docx]

**Supplementary Table 2. Overview of the fixed differences in the 3'UTR of *PRNP* gene between cattle and buffalo.**

| **Cattle** | | |  | **Buffalo** | |
| --- | --- | --- | --- | --- | --- |
| **Position ^a^** | **Position ^b^** | **Allele** |  | **Position ^c^** | **Allele** |
| g.66376 | 4 | G |  | 4 | A |
| g.66459 | 87 | T |  | 87 | C |
| g.66569 | 197 | T |  | 197 | G |
| g.66635 | 263 | G |  | 263 | C |
| g.66647 | 275 | C |  | 275 | T |
| g.66716 | 344 | G |  | 344 | A |
| g.67007 | 635 | G |  | 635 | A |
| g.67045 | 673 | T |  | 673 | G |
| g.67047 | 675 | A |  | 675 | C |
| g.67157-67159 | 785-787 | AAA |  | 780 | del |
| g.67192 | 820 | C |  | 812 | A |
| g.67211 | 839 | G |  | 831 | A |
| g.67310 | 938 | A |  | 930 | G |
| g.67350 | 978 | del |  | 970-997 | GATGCTGGGAAAAACTGAAGGCAGGAGG |
| g.67359 | 987 | T |  | 1007 | C |
| g.67360 | 988 | G |  | 1008 | C |
| g.67373 | 1001 | A |  | 1021 | C |
| g.67394 | 1022 | T |  | 1042 | C |
| g.67440 | 1068 | del |  | 1088-1089 | AG |
| g.67450 | 1078 | C |  | 1100 | T |
| g.67451 | 1079 | A |  | 1101 | G |
| g.67465 | 1093 | G |  | 1115 | A |
| g.67473 | 1101 | A |  | 1123 | G |
| g.67478 | 1106 | T |  | 1128 | C |
| g.67479 | 1107 | G |  | 1129 | A |
| g.67493 | 1121 | G |  | 1143 | C |
| g.67575 | 1203 | T |  | 1221 | G |
| g.67604 | 1232 | T |  | 1250 | G |
| g.67612 | 1240 | A |  | 1258 | G |
| g.67651 | 1279 | G |  | 1297 | T |
| g.67683 | 1311 | del |  | 1329-1330 | CC |
| g.67718 | 1346 | T |  | 1366 | A |
| g.67781 | 1409 | G |  | 1429 | A |
| g.67794 | 1422 | A |  | 1442 | C |
| g.67825 | 1453 | G |  | 1473 | C |
| g.67856 | 1484 | G |  | 1504 | A |
| g.67893 | 1521 | G |  | 1541 | A |
| g.67934 | 1562 | A |  | 1582 | G |
| g.67963 | 1591 | G |  | 1611 | A |
| g.68075 | 1703 | C |  | 1723 | T |
| g.68085 | 1713 | C |  | 1733 | A |
| g.68087 | 1715 | C |  | 1735 | G |
| g.68101 | 1729 | A |  | 1749 | G |
| g.68130 | 1758 | G |  | 1778 | A |
| g.68138 | 1766 | T |  | 1786 | C |
| g.68141 | 1769 | G |  | 1789 | A |
| g.68236 | 1864 | T |  | 1884 | C |
| g.68305 | 1933 | T |  | 1953 | C |
| g.68355 | 1983 | C |  | 2003 | A |
| g.68356 | 1984 | del |  | 2004-2011 | ATAAAAAA |
| g.68432 | 2060 | A |  | 2088 | T |
| g.68459 | 2087 | A |  | 2115 | G |
| g.68587 | 2215 | C |  | 2243 | T |
| g.68596 | 2224 | C |  | 2252 | G |
| g.68652 | 2280 | C |  | 2308 | T |
| g.68654 | 2282 | T |  | 2310 | C |
| g.68655 | 2283 | G |  | 2311 | A |
| g.68772 | 2400 | C |  | 2428 | del |
| g.68778 | 2406 | C |  | 2433 | del |
| g.68808 | 2436 | T |  | 2462 | C |
| g.68849 | 2477 | T |  | 2502 | A |
| g.68856 | 2484 | C |  | 2509 | A |
| g.68875 | 2503 | G |  | 2528 | A |
| g.68902 | 2530 | A |  | 2555 | G |
| g.68930 | 2558 | G |  | 2583 | C |
| g.68962 | 2590 | G |  | 2615 | A |
| g.68966 | 2594 | T |  | 2619 | C |
| g.68967 | 2595 | G |  | 2620 | A |
| g.68971 | 2599 | A |  | 2624 | C |
| g.69074 | 2702 | T |  | 2727 | C |
| g.69100 | 2728 | C |  | 2753 | del |
| g.69109 | 2737 | G |  | 2761 | del |
| g.69110 | 2738 | C |  | 2761 | del |
| g.69112 | 2740 | T |  | 2762 | C |
| g.69133 | 2761 | C |  | 2783 | T |
| g.69134 | 2762 | A |  | 2784 | G |
| g.69148 | 2776 | C |  | 2798 | T |
| g.69185 | 2813 | C |  | 2835 | T |
| g.69197 | 2825 | del |  | 2847 | A |
| g.69247 | 2875 | C |  | 2898 | A |
| g.69251 | 2879 | A |  | 2902 | G |
| g.69287 | 2915 | A |  | 2938 | G |
| g.69300 | 2928 | C |  | 2951 | A |
| g.69325 | 2953 | T |  | 2976 | G |
| g.69359 | 2987 | G |  | 3010 | A |
| g.69367 | 2995 | A |  | 3018 | G |
| g.69383 | 3011 | T |  | 3034 | C |
| g.69401 | 3029 | C |  | 3052 | A |
| g.69431 | 3059 | T |  | 3082 | C |
| g.69440 | 3068 | G |  | 3091 | A |
| g.69482 | 3110 | C |  | 3133 | T |
| g.69504 | 3132 | C |  | 3155 | T |
|  |  |  |  |  |  |

Positions a, b and c indicate positions in sequence AJ298878, KY189378 and KY189403 of GeneBank, respectively.
