# Supplementary material for: Fixed differences in the 3′UTR of buffalo PRNP gene provide binding sites for miRNAs post-transcriptional regulation
Source: Oncotarget. 2017 May 2;8(28):46006–19. doi: 10.18632/oncotarget.17545 (PMC5542244; doi:10.18632/oncotarget.17545)
Supplement: Supplementary file 3 [file oncotarget-08-46006-s003.docx]

**Supplementary Table 3: Target segment fixed point mutation of *PRNP* 3'UTR.**

| **Name of the sequence** | **Sequence（5’→3’)** | **Position** ^a^ | **Length (bp)** |
| --- | --- | --- | --- |
| UTR-1 up | TCGAGGAAGGCAGGAGGGATGCTGGGAAAAACTGAAGGCAGGAGGAGAAGGGGACCACAGAGGATGAGGC | 958-  1020 | 70 |
| UTR-1 down | GGCCGCCTCATCCTCTGTGGTCCCCTTCTCCTCCTGCCTTCAGTTTTTCCCAGCATCCCTCCTGCCTTCC |  | 70 |
| UTR-1m up | TCGAGGAAGGCAGGAGGGAGGATTGGAACACCCGATGGCAGGAGGAGAAGGGGACCACAGAGGATGAGGC |  | 67 |
| UTR-1m down | GGCCGCCTCATCCTCTGTGGTCCCCTTCTCCTCCTGCCATCGGGTGTTCCAATCCTCCCTCCTGCCTTCC |  | 67 |
| UTR-2 up | TCGAGCTCCAGGAGTTGGCAATCGACAGGGAGTCCTGGTGTCCTGCAGTCCATGC | 1067-1114 | 55 |
| UTR-2 down | GGCCGCATGGACTGCAGGACACCAGGACTCCCTGTCGATTGCCAACTCCTGGAGC |  | 55 |
| UTR-2m up | TCGAGCTCCAGGAGTTGGCAATCGACCGGTAGTCCTGGTGTCCTGCAGTCCATGC |  | 55 |
| UTR-2m down | GGCCGCATGGACTGCAGGACACCAGGACTACCGGTCGATTGCCAACTCCTGGAGC |  | 55 |
| UTR-3 up | TCGAGGTCAAAAAACAAAATTAGGTCCTTGGTTACTGTAAAATTAACTTTTGAGC | 1338-1385 | 55 |
| UTR-3 down | GGCCGCTCAAAAGTTAATTTTACAGTAACCAAGGACCTAATTTTGTTTTTTGACC |  | 55 |
| UTR-3m up | TCGAGGTCAAAAAACAAAATTAGGTCCTTGGTTTCCGCAAAATTAACTTTTGAGC |  | 55 |
| UTR-3m down | GGCCGCTCAAAAGTTAATTTTGCGGAAACCAAGGACCTAATTTTGTTTTTTGACC |  | 55 |

^a^ Position indicate position in sequence KY189403 of the 3'UTR of *PRNP* in buffalo.
